# Results of the UK NEQAS for Molecular Genetics reference sample analysis

**DOI:** 10.1136/jclinpath-2018-205277

**Published:** 2018-07-20

**Authors:** Susan D Richman, Jennifer Fairley, Jacqueline A Hall, Nakul Nataraj, Mrudul Bhide, Aron Lau, Kara L Norman, Zandra C Deans

**Affiliations:** 1 Department of Pathology and Tumour Biology, Leeds Institute of Cancer and Pathology, St James University Hospital, Leeds, UK; 2 UK NEQAS for Molecular Genetics, Department of Laboratory Medicine, The Royal Infirmary, Edinburgh, UK; 3 Thermo Fisher Scientific, Fremont, California, USA

**Keywords:** EQA, colorectal cancer, lung cancer, melanoma

## Abstract

**Aims:**

In addition to providing external quality assessment (EQA) schemes, United Kingdom National External Quality Assessment service (UK NEQAS) for Molecular Genetics also supports the education of laboratories. As an enhancement to the Molecular Pathology EQA scheme, a human cell-line reference sample, manufactured by Thermo Fisher Scientific (AcroMetrix), was provided for analysis. This contained many variants, present at frequencies between 1% and 17.9%.

**Methods:**

One hundred and one laboratories submitted results, with a total of 2889 test results on 53 genes being reported. Known polymorphisms, 46/2889 (1.59%) results, were excluded. Variants detected in the seven most commonly reported (and clinically relevant) genes, *KRAS, NRAS, BRAF, EGFR, PIK3CA, KIT* and *PDGFRA*, are reported here, as these genes fall within the scope of UK NEQAS EQA schemes.

**Results:**

Next generation sequencing (NGS) was the most commonly performed testing platform. There were between 5 and 27 validated variants in the seven genes reported here. Eight laboratories correctly reported all five *NRAS* variants, and two correctly reported all eight *BRAF* variants. The validated mean variant frequency was lower than that determined by participating laboratories, with single-gene testing methodologies showing less variation in estimated frequencies than NGS platforms. Laboratories were more likely to correctly identify clinically relevant variants.

**Conclusions:**

Over 100 laboratories took the opportunity to test the ‘educational reference sample’, showing a willingness to further validate their testing platforms. While it was encouraging to see that the most widely reported variants were those which should be included in routine testing panels, reporting of variants was potentially open to interpretation, thus clarity is still required on whether laboratories selectively reported variants, by either clinical relevance or variant frequency.

## Introduction

Having moved well into the era of personalised medicine, laboratories are being called on to routinely test solid tumours, such a colorectal,^1–5^ lung,^6–9^ melanoma^10^ and gastrointestinal stromal tumours (GIST)^11–13^ for a variety of biomarkers, which will determine treatment options.

One way to monitor accuracy and reproducibility of testing is through participation in external quality assessment (EQA) schemes. Several studies have demonstrated the benefits of scheme participation,^14–18^ having seen an improvement that is, a decrease in genotyping errors, with continuous EQA participation.

The United Kingdom National External Quality Assessment service (UK NEQAS) for Molecular Genetics has been providing EQA schemes for the assessment of colorectal cancer (CRC) (since 2009), lung cancer (since 2010), melanoma (since 2012) and GIST (since 2008). The scheme sends tumour samples to participating laboratories 2–3 times per year and provides an assessment of genotyping, interpretation and clerical accuracy. These molecular pathology EQAs are ISO 17043 (https://www.iso.org/standard/29366.html) accredited.

In addition to the samples sent out as part of run 2 of the 2015–16 Molecular Pathology EQA schemes, a manufactured human cell-line reference sample (Thermo Fisher Scientific (AcroMetrix)) was also distributed to laboratories. Here we report on the data gathered from 101 laboratories, who returned genotyping and variant allele frequency results.

## Materials and methods

UK NEQAS for Molecular Genetics supplied an educational EQA case using a reference sample as part of the Molecular Pathology 2015–2016 run 2 distributions. One rolled section (10 µm thickness) of formalin-fixed paraffin-embedded (FFPE) cell-line material was provided to each laboratory. This was manufactured by Thermo Fisher Scientific (AcroMetrix), to contain a large number of variants, present at varying frequencies. These frequencies were independently validated by digital droplet PCR prior to distribution.

Laboratories participating in the CRC, lung cancer, melanoma and GIST Molecular Pathology EQA schemes were invited, as a voluntary exercise, to extract DNA from the FFPE section and test using their routine protocols for any of the targets in table 1.

**Table 1:** Genes which could be tested for the presence of variants in the reference sample

ABL1	EGFR	GNA11	KIT	PDGFRA	VHL
AKT1	ERBB2	GNAQ	KRAS	PIK3CA	TP53
ALK	ERBB4	GNAS	MAP2K1	PTEN	
APC	EZH2	HNF1A	MET	PTPN11	
ATM	FBXW7	HRAS	MLH1	RB1	
BRAF	FGFR1	IDH1	MPL	RET	
CDH1	FGFR2	IDH2	MSH6	SMAD4	
CDKN2A	FGFR3	JAK2	NOTCH1	SMARCB1	
CSF1R	FLT3	JAK3	NPM1	SMO	
CTNNB1	FOXL2	KDR	NRAS	STK11	

Results were submitted using a supplied *proforma*, which also requested information on test methodology and the percentage variant frequency detected for each variant reported.

No EQA scores were assigned to participating laboratories, and the Scheme subsequently provided these laboratories with a complete breakdown of the validated variant frequencies within each gene, thus allowing the reassessment of raw data, if variants had not been identified by participants.

## Results

### Participation

**Figure 1:**
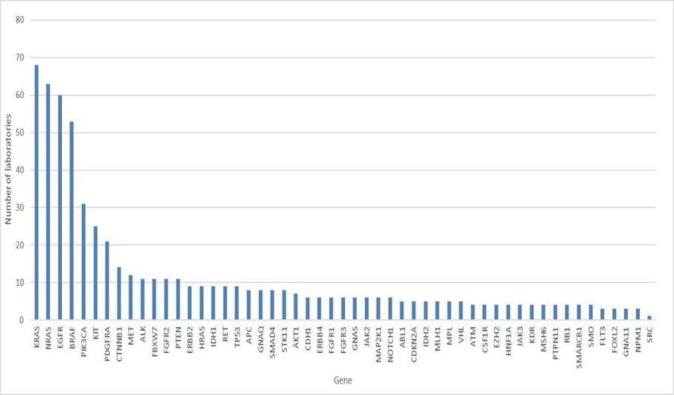
Genes tested by participating laboratories.

One hundred and one laboratories tested this reference sample and submitted results. Several laboratories carried out more than one test methodology and therefore reported multiple results. A total of 2889 test results were reported spanning 53 different genes. There were only 52 different genes in the reference sample; however, one laboratory unexpectedly reported test results for proto-oncogene tyrosine-protein kinase Src (SRC). Known polymorphisms, which accounted for 1.59% of results, were excluded from subsequent analysis. Fifty-eight variants present in the reference sample were incorrectly reported as ‘no mutation detected’, spread over 17 different genes. The analysis reported here is confined to the seven most commonly tested genes: *KRAS, NRAS, BRAF, EGFR, PIK3CA, KIT* and *PDGFRA* (figure 1).

### Testing methodologies

The most commonly used mutation test was by next generation sequencing (NGS), with 48/101 (47.5%) of laboratories returning data using NGS. The percentage of results reported by NGS, compared with single-gene technologies (pyrosequencing, Sanger sequencing, mass spectrometry, real-time PCR, StripAssay, EntroGen mutation kits, Cobas 4800, LightMix Kit) is shown in table 2. The Ion Torrent was the preferred NGS platform for the testing of all genes, with the Illumina platform being the second most common. Data provided by participants relating to the panel used were sparse, but most commonly reported was the AmpliSeq for Illumina Cancer Hotspot Panel V.2.

**Table 2:** Percentage of all results submitted which were obtained by NGS

Gene	Number of results reported by NGS	Percentage of the total number of results reported by NGS (%)
*BRAF*	115	56.1
*EGFR*	298	81.4
*KIT*	143	59.1
*KRAS*	115	56.1
*NRAS*	128	59
*PDGFRA*	84	92.3
*PIK3CA*	230	94.3

NGS, next generation sequencing.

### Variants and variant allele frequencies

The number of validated variants within each of the seven most commonly tested genes is shown in table 3, along with the mean validated frequency. Digital droplet PCR was used by Thermo Fisher to independently validate the variant frequencies within the reference samples prior to distribution. Multiple measurements were taken and an average result determined.

**Table 3:** Validated variant numbers and frequencies within the seven most commonly reported genes

Gene	Number of variants present	Mean variant frequency (%)
*BRAF*	8	2.6
*EGFR*	27	2.6
*KIT*	24	4.2
*KRAS*	8	4.3
*NRAS*	5	7.4
*PDGFRA*	8	4.2
*PIK3CA*	24	4.7

In terms of the correct reporting of all variants in each gene, only eight laboratories reported all five variants present in *NRAS* and two laboratories correctly identified all of the eight variants in *BRAF*. No laboratories correctly reported all the variants present in any of the other genes. There were in fact several laboratories who failed to report the presence of any variants in the seven most commonly reported genes and returned a wild type (WT) result. These are detailed in table 4.

**Table 4:** The number of laboratories incorrectly reporting a WT result

Gene	Mean variant frequency (%)	Number of laboratories reporting WT result	Number of tests performed with no variants reported	Methodologies employed (n)
*BRAF*	2.6	21	22	Sanger (4), Pyrosequencing (4), Cobas 4800 (5), RT-PCR (2), HRM (2)*
*EGFR*	2.6	3	6	Sanger (1), Pyrosequencing (1), NGS/Ion Torrent (2), RT-PCR (1)
*KIT*	4.2	1	1	Sanger (1)
*KRAS*	4.3	7	9	Sanger (4), Pyrosequencing (2), NGS/Ion Torrent (1), RT-PCR (1), Entrogen (1)
*NRAS*	7.4	3	5	Sanger† (2), Pyrosequencing (2), NGS/Ion Torrent (1)
*PDGFRA*	4.2	1	1	Sanger (1)
*PIK3CA*	4.7	1	2	Pyrosequencing (1), NGS/Ion Torrent (1)

*In addition to those listed in the table, one laboratory used Therascreen, one laboratory used Amoy Dx BRAF Mutation Detection kit, one laboratory used Idylla, one laboratory performed NGS on an Ion Torrent platform and one laboratory did not disclose the NGS methodology used.

†Sanger sequencing.

WT, wild type.

More variants were engineered into the reference sample than are clinically actionable, which may account for the differences seen in variant reporting. The most commonly reported variant in each gene is shown in table 5, along with the clinically relevant variants engineered into the sample.

**Table 5:** Most commonly reported variants in each gene and clinically relevant mutations

Gene	Most commonly reported variant by participants	Clinically relevant variants known to be present in the sample
*BRAF*	c.1799T>A p.(Val600Glu)	c.1799T>A p.(Val600Glu)
*EGFR*	c.2235_2249del p.(Glu746_Ala750del)	c.2156G>C p.(Gly719Ala); c.2235_2249del p.(Glu746_Ala750del); c.2573T>G p.(Leu858Arg) and c.2582T>A p.(Leu861Gln)
*KIT*	c.1727T>C p.(Leu576Asn)	c.1504_1509dup p.(Ala502_Tyr503dup) and c.1727T>C p.(Leu576Asn)
*KRAS*	c.35G>A p.(Gly12Asp)	c.35G>A p.(Gly12Asp), c.175G>A p.(Ala59Thr) c.183A>C p.(Gln61His) and c.351A>C p.(Lys117Asn)
*NRAS*	c.35G>A p.(Gly12Asp)	c.35G>A p.(Gly12Asp) and c.182A>G p.(Gln61Arg)
*PDGFRA*	c.1698_1712del and c.2525A>T p.(Asp842Val)	c.2525A>T p.(Asp842Val)
*PIK3CA*	c.3140A>G p.(His1047Arg)	c.1624G>A p.(Glu542Lys); c.1633G>A p.(Glu545Lys) and c.3140A>G p.(His1047Arg)

For *NRAS*, 46/63 (73%) and 44/63 (69.8%) of laboratories, respectively, correctly detected the two clinically relevant variants; c.35G>A and c.182A>G. There were four clinically relevant variants in *KRAS* (see table 5) and these were identified by 47/68 (69.1%), 25/68 (36.8%), 37/68 (54.4%) and 28/68 (41.2%) laboratories, respectively.

The reference sample included the four most common *EGFR* mutations associated with sensitivity to EGFR receptor tyrosine kinase inhibitors (TKIs): c.2156G>C, c.2235_2249del, c.2573T>G and c.2582T>A. These were correctly reported by 24/59 (40.7%), 50/59 (84.7%), 26/59 (44.1%) and 22/59 (37.3%) laboratories, respectively. Two laboratories reported unexpected variants, using the Ion Torrent AmpliSeq in-house gene panel and an Illumina MiSeq custom panel.

The c.1799T>A mutation is the most common *BRAF* variant in melanoma and colorectal tumours, and so would be expected to be included in testing panels. Thirty-two of the 53 laboratories (60.4%) correctly reported the detection of this variant. Surprisingly, 39.6% of laboratories failed to do so.

Exons 10 and 21 are known *PIK3CA* mutation hotspots, and 3 of the 24 variants in the reference sample are contained within these exons (see table 5). Of the 31 laboratories reporting a result for *PIK3CA* gene variant testing, the variant were correctly identified by 17/31 (54.8%), 18/31 (58.1%) and 26/319 (83.9%) laboratories (respectively). Two laboratories, both using the Qiagen GeneRead Clinically Relevant Mutations panel run on an Illumina MiSeq NGS platform, reported unexpected variants in *PIK3CA*. These were not reported by any other laboratory.

The *KIT* gene variants within exons 9 and 11, including c.1504_1509dup and c.1727T>A were correctly identified by 17/25 (68%) and 18/25 (72%) laboratories, respectively. Two laboratories reported unexpected variants using the Ion Torrent AmpliSeq inhouse panel and the Illumina MiSeq with the Qiagen GeneRead Clinically relevant mutations panel.

For *PDGFRA*, the most commonly reported mutation was c.2525A>T, which confers resistance to TKIs. This was identified by 14/21 (66.7%) laboratories, along with c.1698_1712del1712, also by 14/21 (66.7%) laboratories.

### Accuracy of testing for the presence of variants

**Figure 2:**
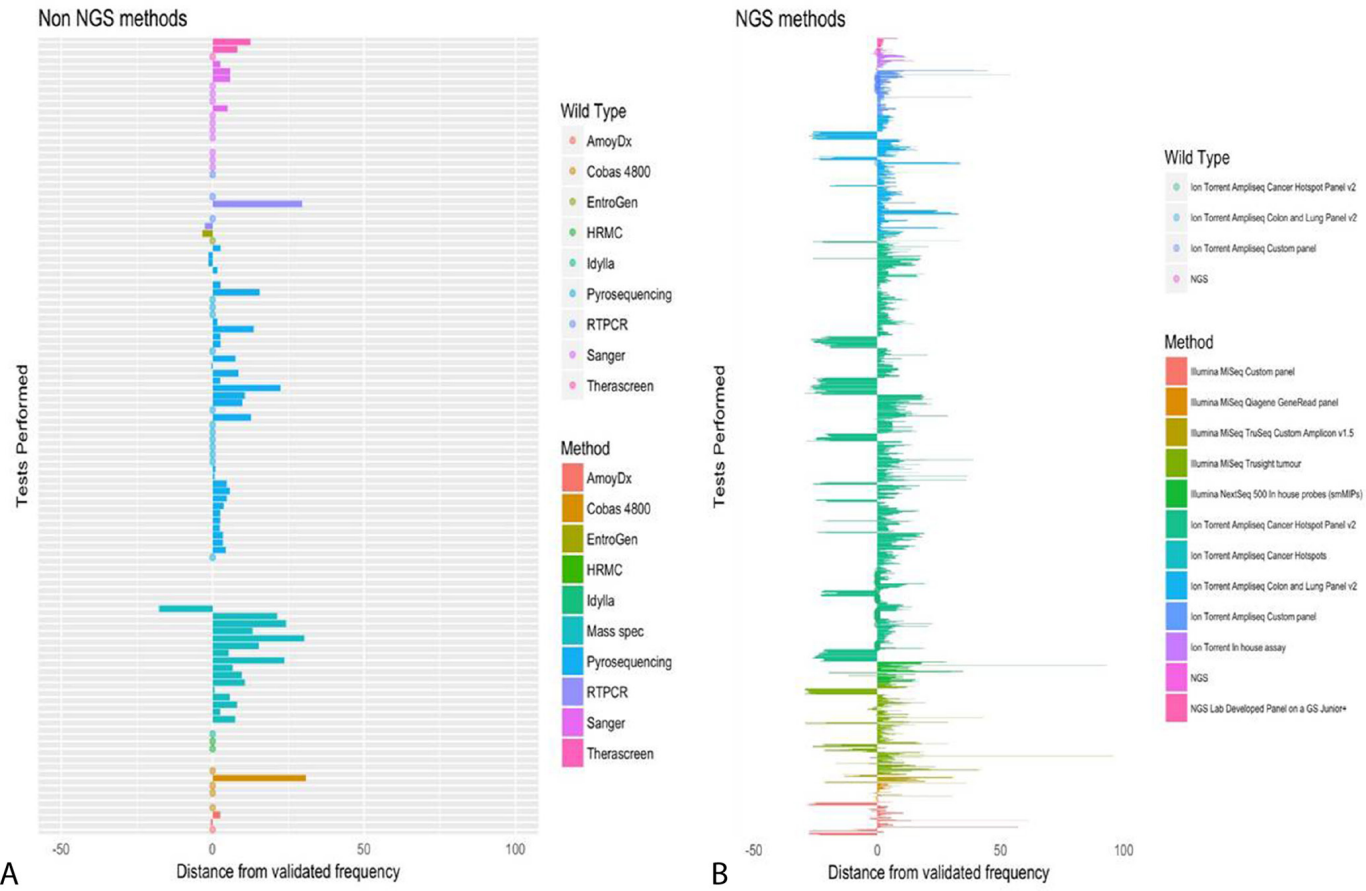
Variant frequencies reported by participating laboratories, using NGS platforms. The bars represent the percentage variation (+/–) from the validated test allelic frequency. Bars are grouped and coloured by test method. Left panel, non-NGS methodologies; Right panel, NGS methodologies. NGS, next generation sequencing.

As each laboratory was required to report the percentage variant frequency, it was possible to calculate a mean and median result for each gene. For the purposes of this analysis, the reported frequencies have been grouped by gene, and thus a range for mean and median is shown (table 6). As can be clearly seen, laboratories consistently overestimated the variant allele frequency, particularly for variants in *PIK3CA, KIT* and *PDGFRA. BRAF* was the most accurately reported, in terms of mean and median frequencies. When the test methodology was taken into account, less variation was observed in single-gene testing, as opposed to NGS platforms (figure 2), but nonetheless, there was still an overestimation.

**Table 6:** Calculated mean and median variant frequencies, from data submitted by each laboratory

Gene	Validated mean variant frequency (%)	Range of participant reported mean variant frequencies (%)	Range of participant reported median variant frequencies (%)
*BRAF*	2.6	4.13–8.21	4.10–5.00
*EGFR*	2.6	3.62–8.84	4.00–7.59
*KIT*	4.2	7.00–17.00	5.99–17.40
*KRAS*	4.3	5.71–10.55	6.06–9.09
*NRAS*	7.4	11.85–16.16	11.43–15.74
*PDGFRA*	4.2	9.37–15.00	8.18–11.22
*PIK3CA*	4.7	10.00–17.80	9.41–17.00

## Discussion

With the concept of personalised medicine expanding rapidly into practice and biomarker screening becoming routine on many laboratories, it is more important than ever that laboratories reporting these results can demonstrate high quality, accurate validated assays. As results from biomarker screening often determine patient treatment, there must be a high degree of confidence in the assay delivery.

One way that laboratories can demonstrate this is through participation in an EQA scheme, such as those developed, administered and regularly provided by UK NEQAS. In addition to the biannual EQA samples distributed through the CRC, GIST, lung cancer and melanoma schemes, laboratories also received an educational manufactured human cell-line reference sample. A complete set of validated variant and corresponding frequencies was provided to each participating laboratory following result submission to UK NEQAS.

It was reassuring to see that for the most part, the most frequently reported variants were those with clinical relevance. In terms of the variants of relevance to CRC, over two-thirds of laboratories reported the two mutations in *NRAS*, and between 36.8% and 69% correctly identified the clinically relevant variants in *KRAS*. The variant allele frequency of the *NRAS* variants was 7.4%, compared with 4.3% for the *KRAS* variants, which may have explained the lower level of detection for the latter gene variants. Furthermore, slightly more laboratories used NGS for the detection of *NRAS* mutations, compared with *KRAS*, possibly providing an overall lower limit of detection. We must not exclude the possibility that some laboratories do not report mutant allele frequencies below 5%, and thus did detect, but did not report the *KRAS* variants, which they may have found below their lower limit for reporting. Laboratories were encouraged to review their raw data following release of the validated variant data.

Sixty per cent of laboratories correctly identified the common c.1799T>A p.(V600E) mutation in *BRAF*, in spite of the manufactured variant allele frequency being stated as 2.6%. In CRC screening, *BRAF* is likely to be included in gene panels due to the poor prognosis that this codon 600 mutation confers, and its association with microsatellite instability, both of which will be assessed as part of Lynch Screening (https://www.nice.org.uk/guidance/dg27). In melanoma, the presence of this mutation is indicative of response to anti-EGFR therapy.

The four clinically relevant variants in *EGFR* were identified by very differing percentages of participating laboratories, ranging from 37.3% for c.2582T>A p. (Leu861Gln), up to 84.7% for c.2235_2249del p.(Glu746_Ala750del). This may again be as a result of the very low mean variant allele frequency (2.6%) resulting in laboratories not reporting low level mutations, but the clinical importance of correct mutation identification should be highlighted, as patients whose non-small-cell lung cancers contains such mutations, will fail to respond to TKIs.

Mutation screening of GIST tumours is complex as these tumours arise as a result of a driver mutation in either *KIT* of *PRGFRA*. In addition, there are multiple mutation types, making these tumours difficult to accurately screen. Reassuringly, two thirds of laboratories correctly identified the activating mutation in exon 18 of *PDGFRA*, which confers resistance to TKIs, and again over two thirds correctly identified activating mutations in *KIT.*

Overall, the fact that clinically relevant and actionable variants were reported most frequently, provides reassuring evidence that gene tests have been optimised to detect the majority of clinical important variants in most laboratories.

There were several laboratories which failed to identify any variants in the sample and reported it as WT. With the exception of *KIT* gene testing, Sanger Sequencing was reported as the testing methodology for each gene in at least one laboratory. It is widely acknowledged that the limit of detection for Sanger Sequencing^19^ is 20%–25% mutant allele frequency in a WT allele background, which may explain the failure to detect the variants presence at lower levels.

Twenty-one laboratories did not detect a variant in the *BRAF* gene. There was huge variation in the testing methodologies used and this perhaps demonstrates reduced access to a more sensitive NGS-based tests for variant detection for this gene. *KRAS* was the next most common gene to be reported as WT (by seven laboratories). The Scheme has seen a large increase in laboratories participating for the first time with limited biomarker molecular testing experience. There is a possibility that such laboratories have limited access to high sensitivity sequencing platforms.

We cannot ignore the engineered artificial nature of the sample, as a contributing factor when it comes to incorrect variant detection. Samples usually received as part of the standard EQA schemes are also FFPE in nature, but consist of a single human tumour sample, rather than an engineered FFPE cell-line. Furthermore, as the reference sample consisted of a single tissue FFPE curl, this would not have been a standard sample format for many participating laboratories and thus added in an additional variable to standard sample processing pipelines.

Increasingly, laboratories are being expected to obtain molecular test results from small and often challenging biopsy samples. To meet these challenges, these laboratories must ensure that their assay design has been fully optimised and that the scope of gene testing is sufficient to meet the requirements of the tests.

Having the ability to test samples such as this reference sample and receive the full genotyping results from UK NEQAS should provide high quality validation information and alert laboratories to any suboptimal assays. This should guide reoptimisation to ensure high quality sample screening.

**Take home messages**

- Laboratories carrying out mutation screening must maximise opportunities to enrol in External Quality Assurance schemes.
- Provision of the complete mutation profile of the reference sample to laboratories has allowed, where required, assay optimisation procedures to be undertaken.
- Schemes such as those run by UK NEQAS offer samples for testing and educational support, which must not be overlooked and should be embraced.

## References

[1] Amado RG, Wolf M, Peeters M, et al Wild-type KRAS is required for panitumumab efficacy in patients with metastatic colorectal cancer. J Clin Oncol 2008;26:1626–34. 10.1200/JCO.2007.14.7116 18316791

[2] Bokemeyer C, Bondarenko I, Hartmann JT, et al Efficacy according to biomarker status of cetuximab plus FOLFOX-4 as first-line treatment for metastatic colorectal cancer: the OPUS study. Ann Oncol 2011;22:1535–46. 10.1093/annonc/mdq632 21228335

[3] Douillard JY, Siena S, Cassidy J, et al Randomized, phase III trial of panitumumab with infusional fluorouracil, leucovorin, and oxaliplatin (FOLFOX4) versus FOLFOX4 alone as first-line treatment in patients with previously untreated metastatic colorectal cancer: the PRIME study. J Clin Oncol 2010;28:4697–705. 10.1200/JCO.2009.27.4860 20921465

[4] Peeters M, Price TJ, Cervantes A, et al Randomized phase III study of panitumumab with fluorouracil, leucovorin, and irinotecan (FOLFIRI) compared with FOLFIRI alone as second-line treatment in patients with metastatic colorectal cancer. J Clin Oncol 2010;28:4706–13. 10.1200/JCO.2009.27.6055 20921462

[5] Van Cutsem E, Köhne CH, Hitre E, et al Cetuximab and chemotherapy as initial treatment for metastatic colorectal cancer. N Engl J Med 2009;360:1408–17. 10.1056/NEJMoa0805019 19339720

[6] Fukuoka M, Wu YL, Thongprasert S, et al Biomarker analyses and final overall survival results from a phase III, randomized, open-label, first-line study of gefitinib versus carboplatin/paclitaxel in clinically selected patients with advanced non-small-cell lung cancer in Asia (IPASS). J Clin Oncol 2011;29:2866–74. 10.1200/JCO.2010.33.4235 21670455

[7] Lynch TJ, Bell DW, Sordella R, et al Activating mutations in the epidermal growth factor receptor underlying responsiveness of non-small-cell lung cancer to gefitinib. N Engl J Med 2004;350:2129–39. 10.1056/NEJMoa040938 15118073

[8] Paez JG, Jänne PA, Lee JC, et al EGFR mutations in lung cancer: correlation with clinical response to gefitinib therapy. Science 2004;304:1497–500. 10.1126/science.1099314 15118125

[9] Pao W, Miller V, Zakowski M, et al EGF receptor gene mutations are common in lung cancers from "never smokers" and are associated with sensitivity of tumors to gefitinib and erlotinib. Proc Natl Acad Sci U S A 2004;101:13306–11. 10.1073/pnas.0405220101 15329413 PMC516528

[10] Chapman PB, Hauschild A, Robert C, et al Improved survival with vemurafenib in melanoma with BRAF V600E mutation. N Engl J Med 2011;364:2507–16. 10.1056/NEJMoa1103782 21639808 PMC3549296

[11] Haller F, Schulten HJ, Armbrust T, et al Multicentric sporadic gastrointestinal stromal tumors (GISTs) of the stomach with distinct clonal origin: differential diagnosis to familial and syndromal GIST variants and peritoneal metastasis. Am J Surg Pathol 2007;31:933–7. 10.1097/01.pas.0000213440.78407.27 17527083

[12] Lasota J, Miettinen M Clinical significance of oncogenic KIT and PDGFRA mutations in gastrointestinal stromal tumours. Histopathology 2008;53:245–66. 10.1111/j.1365-2559.2008.02977.x 18312355

[13] Wong NA Gastrointestinal stromal tumours--an update for histopathologists. Histopathology 2011;59:807–21. 10.1111/j.1365-2559.2011.03812.x 21668468

[14] Deans ZC, Bilbe N, O’Sullivan B, et al Improvement in the quality of molecular analysis of EGFR in non-small-cell lung cancer detected by three rounds of external quality assessment. J Clin Pathol 2013;66:319–25. 10.1136/jclinpath-2012-201227 23378269

[15] Deans ZC, Tull J, Beighton G, et al Molecular genetics external quality assessment pilot scheme for KRAS analysis in metastatic colorectal cancer. Genet Test Mol Biomarkers 2011;15:777–83. 10.1089/gtmb.2010.0239 21851273

[16] Deans ZC, Wallace A, O’Sullivan B, et al External quality assessment of BRAF molecular analysis in melanoma. J Clin Pathol 2014;67:120–4. 10.1136/jclinpath-2013-201848 24098023

[17] Richman SD, Fairley J, Butler R, et al RAS screening in colorectal cancer: a comprehensive analysis of the results from the UK NEQAS colorectal cancer external quality assurance schemes (2009-2016). Virchows Arch 2017;471:721–9. 10.1007/s00428-017-2162-7 28653203 PMC5711992

[18] Wong NA, Deans ZC, Ramsden SC The UK NEQAS for Molecular Genetics scheme for gastrointestinal stromal tumour: findings and recommendations following four rounds of circulation. J Clin Pathol 2012;65:786–90. 10.1136/jclinpath-2012-200851 22685257

[19] Sanger F, Nicklen S, Coulson AR DNA sequencing with chain-terminating inhibitors. Proc Natl Acad Sci U S A 1977;74:5463–7. 10.1073/pnas.74.12.5463 271968 PMC431765

